# Mirror Neurons and Pain: A Scoping Review of Experimental, Social, and Clinical Evidence

**DOI:** 10.3390/healthcare14020280

**Published:** 2026-01-22

**Authors:** Marco Cascella, Pierluigi Manchiaro, Franco Marinangeli, Cecilia Di Fabio, Giacomo Sollecchia, Alessandro Vittori, Valentina Cerrone

**Affiliations:** 1 Department of Medicine, Surgery and Dentistry “Scuola Medica Salernitana”, University of Salerno, 84081 Salerno, Italy; 2 Surgical Pain Therapy Centre, MediClinic Hospital, 35043 Padua, Italy; 3 Department of Life, Health and Environmental Sciences (MeSVA), University of L’Aquila, Piazzale Salvatore Tommasi 1, Blocco 11, 67010 L’aquila, Italy; 4 Department of Anesthesia, Critical Care and Pain Medicine, ARCO, Ospedale Pediatrico Bambino Gesù IRCCS, Piazza S. Onofrio 4, 00165 Rome, Italy

**Keywords:** pain, mirror neuron system, scoping review, phantom limb, mirror therapy

## Abstract

**Background:** The mirror neuron system (MNS) has been proposed as a key neural mechanism linking action perception, motor representation, and social cognition. This framework has increasingly been applied to pain research, encompassing pain empathy, observational learning of pain, and rehabilitative interventions such as mirror therapy. However, the literature is conceptually heterogeneous, methodologically diverse, and spans experimental, social, and clinical domains. **Objective:** This scoping review aims to map the extent, nature, and characteristics of the available evidence on the relationship between the MNS and pain, clarifying how MNS-related mechanisms are defined, investigated, and applied across different contexts. **Methods:** A scoping review was conducted using the methodological framework proposed by the Joanna Briggs Institute and reported in accordance with PRISMA-ScR guidelines. We searched PubMed/MEDLINE, Scopus, Web of Science, and PsycINFO. Studies were included if they addressed MNS-related mechanisms in pain processing, pain empathy, pain modulation, or pain rehabilitation. Eligible studies were charted and synthesized descriptively and thematically. **Results:** Twenty-one studies met the inclusion criteria. The evidence was predominantly derived from clinical and rehabilitative settings, with most studies focusing on mirror therapy or mirror visual feedback interventions. The majority of included populations consisting of adults with chronic pain conditions, particularly phantom limb pain and complex regional pain syndrome. Pain intensity, assessed mainly through self-reported clinical scales, was the most frequently reported outcome. A smaller number of studies investigated action observation or motor imagery paradigms, primarily in chronic musculoskeletal pain, showing short-term hypoalgesic effects. Across studies, substantial heterogeneity was observed in the conceptualization of MNS-related constructs, intervention protocols, outcome measures, and follow-up duration. **Conclusions:** Despite extensive theoretical discussion of the MNS, empirical applications are largely confined to clinical mirror-based interventions, with limited use of direct neurophysiological or neuroimaging markers. Since crucial conceptual and methodological gaps constrain comparability and translation into clinical practice, there is a need for clearer operational definitions and more integrated experimental and clinical research approaches.

## 1. Introduction

Pain is a multidimensional experience shaped by nociceptive input combined with cognitive, affective, and social processes [1]. Beyond individual perception, humans can understand, anticipate, and even modulate pain through social interactions, observation of others, and action-related representations [2,3]. In this context, the observation–action matching system, also referred to as the mirror neuron system (MNS), has been proposed as a neural substrate linking perception and action, supporting shared experiences such as action understanding, imitation, embodied simulation, and broader aspects of social cognition [4].

First described in the premotor and parietal cortices of non-human primates, mirror neurons fire during the execution of a goal-directed action and when observing the same action performed by others, suggesting a mechanism for action understanding and learning through perception–action coupling [5]. In humans, converging neuroimaging and neurophysiological evidence supports the existence of a distributed mirror-related network involving inferior frontal, inferior parietal, and sensorimotor regions [4,6].

In addition to motor representations, the MNS has been discussed within broader theoretical frameworks addressing higher-order cognitive and conscious processes, including awareness, understanding, empathy, and grounded cognition. From this perspective, mirror-related mechanisms have been proposed to contribute to the integration of sensory, motor, and contextual information that underlies embodied representations of the self and others [7]. Notably, alterations in these processes have been examined in neurodevelopmental conditions, such as autism spectrum disorders, further supporting the relevance of mirror-related networks for social cognition [8,9].

**Figure 1:**
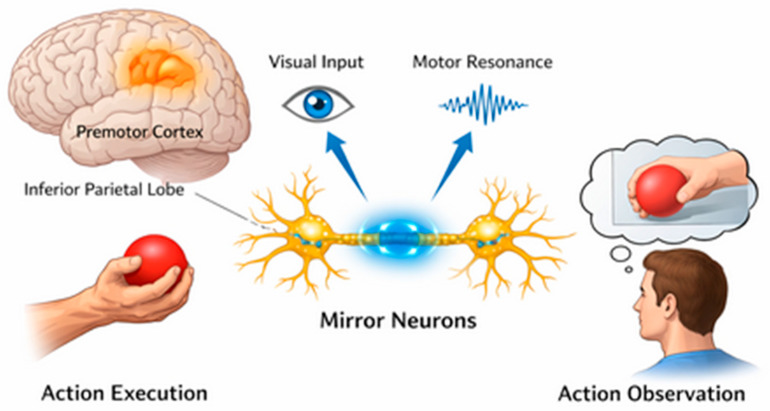
Schematic representation of the mirror neuron system (MNS). Mirror neurons are classically described within a predominantly contralateral fronto-parietal network, including the premotor cortex and inferior parietal lobule. These neurons are activated both during the execution of a goal-directed action and during the observation of the same action performed by others. Visual input related to action observation is integrated with motor representations, giving rise to sensorimotor resonance and action–perception coupling mechanisms that are thought to support action understanding, imitation, and embodied simulation.

Although these broader conceptualizations extend beyond pain research per se, they provide an important theoretical background for interpreting MNS-related frameworks applied to pain, particularly in domains involving empathy, observation, and social modulation of pain experience. Interestingly, within pain research, MNS-related concepts have been applied in different and partially overlapping fields. For example, studies on pain empathy have shown that observing others in pain can activate brain regions involved in affective and, in part, sensorimotor processing, suggesting the role of embodied simulation and sensorimotor resonance in empathic pain responses [10]. Moreover, experimental paradigms of observational learning and social modulation of pain suggest that pain perception can be shaped by observing others’ pain behaviors or relief, potentially through action–perception coupling and expectation-based mechanisms [11,12]. Furthermore, in clinical and rehabilitative settings, interventions such as mirror therapy, mirror visual feedback, and action observation therapy explicitly rely on action observation and motor representation to modulate pain and functional outcomes, particularly in conditions such as phantom limb pain [13] and complex regional pain syndrome (CPRS) [14]. Core principles of the mirror neuron system are schematically illustrated in Figure 1.

Despite this growing body of literature, the use of the term “mirror neuron system” in pain-related studies is far from uniform. Different studies rely on diverse proxies, such as functional magnetic resonance (fMRI) activation patterns [15] or electroencephalogram (EEG) mu rhythm suppression [16], and often conflate MNS-related mechanisms with broader constructs such as empathy, attention, salience, or expectancy. Moreover, the causal role of mirror mechanisms in pain modulation remains a topic of debate, and the translational relevance of experimental findings to clinical pain management is not always clear [17].

Given this conceptual and methodological heterogeneity, we performed a scoping review to systematically map how mirror neuron-related frameworks have been operationalized in pain research, identify dominant paradigms and outcomes, and highlight gaps requiring further investigation. The aim is to provide an integrative overview of the field and inform future hypothesis-driven and translational studies.

## 2. Materials and Methods

### 2.1. Protocol and Registration

This scoping review was conducted following the methodological framework proposed by the Joanna Briggs Institute [18] and reported in accordance with the Preferred Reporting Items for Systematic Reviews and Meta-analysis Protocols extension for scoping review [19]. The protocol was registered in the collaborative project management tool Open Science Framework (https://osf.io/tgsj6/overview, accessed on 31 December 2025).

### 2.2. Eligibility Criteria

To be included in the review, articles needed to explicitly investigate or discuss MNS-related constructs using experimental, neurophysiological, neuroimaging, or clinical paradigms in relation to pain or pain-related outcomes. We considered publications in peer-reviewed journals and written in English. Only primary empirical studies (quantitative and qualitative) were included.

Concerning exclusion criteria, we excluded articles mentioning mirror neurons only tangentially, without empirical or conceptual linkage to pain-related outcomes. We also excluded editorials, commentaries, and opinion pieces without original data. Review articles were screened for citation tracking but were not included as primary evidence.

### 2.3. Search Strategy

The review was guided by the following research question: “*What is known about how mirror neuron-related frameworks are defined, operationalized, and applied in pain research across experimental, social, and clinical contexts?*”

The review was structured according to the Population-Concept-Context (PCC) framework:

- Population: Human participants (healthy individuals and/or patients with pain conditions).
- Concept: MNS or closely related constructs (e.g., action observation, motor imagery, mirror visual feedback, embodied simulation), explicitly linked to pain, nociception, analgesia, or pain empathy.
- Context: Experimental laboratory studies, social neuroscience paradigms, and clinical or rehabilitative settings.

The search strategy was developed by M.C. and V.C. following an initial exploratory phase aimed at identifying relevant keywords and minimizing noise and false-positive results. Preliminary searches were conducted to identify relevant literature and to examine keyword behavior, with particular attention to terms that could introduce noise or false-positive results. The search strategy combined terms related to the MNS (e.g., “mirror neuron”, “action observation”, “motor imagery”, “mirror therapy”) with pain-related terms (e.g., “pain”, “nociception”, “analgesia”, “pain empathy”). Based on this assessment, Boolean exclusion operators (NOT) were incorporated to progressively narrow the search and exclude records that were not aligned with the scope of the review. This iterative process allowed the refinement of the search strategy to maximize relevance while maintaining comprehensive coverage of the topic. The final search syntax was formulated using a combination of Boolean operators (AND, OR, and NOT) and truncation techniques (e.g., wildcards and quotation marks) and was applied across eight electronic databases. The full search strategy for at least one database is reported in Supplement S1. Reference lists of included articles were manually screened to identify additional relevant studies.

### 2.4. Information Sources

A comprehensive literature search was conducted in the following electronic databases: PubMed/MEDLINE, Scopus, Web of Science, and PsycINFO. The bibliographic search was conducted in November 2025 and covered all records available up to December 2024. In addition, the reference lists of all included studies and relevant reviews were manually screened to identify further eligible sources. No direct contact with study authors was undertaken.

#### Selection of Sources of Evidence

All records retrieved from the searches were imported into reference management software, and duplicates were removed. After duplicate removal, titles and abstracts were independently screened by two reviewers against the eligibility criteria (C.D. and G.S.). Full-text articles were subsequently assessed for eligibility by the same reviewers. Any discrepancies were resolved through discussion, with the involvement of M.C. when consensus could not be reached.

### 2.5. Data Charting Process

Data charting was independently performed by C.D. and G.S. using a standardized extraction form developed a priori. The following variables were extracted from each included study:

- Bibliographic details (author, year, country);
- Study aims and design;
- Population characteristics (sample size, health status);
- Definition and operationalization of mirror neuron system-related constructs;
- Experimental or clinical paradigm (e.g., fMRI, EEG, mirror therapy, action observation);
- Type and context of pain (e.g., experimentally induced pain, chronic pain, pain empathy);
- Outcomes assessed (neural, behavioral, clinical);
- Main findings related to MNS and pain;
- Reported limitations.

When necessary, data were simplified into broader conceptual categories (e.g., grouping different paradigms under “pain empathy” or “clinical interventions”) to facilitate mapping and synthesis. Reference management and data organization were supported by V.C. using dedicated bibliographic software (EndNote 2025, Clarivate Analytics).

### 2.6. Critical Appraisal of Individual Sources of Evidence

In accordance with the PRISMA-ScR guidelines, a formal critical evaluation of the methodological quality of the included studies was not planned or conducted. The main objective of this scoping review was to map the available evidence on interventions based on the MNS in the context of pain, rather than to evaluate the efficacy or methodological robustness of the studies. As a result, the evidence sources were summarized descriptively, without applying weighting criteria based on quality. Additionally, in accordance with the usual methodology for scoping reviews, an assessment of the risk of bias of the included studies was not performed.

### 2.7. Synthesis of Results

Extracted data were synthesized using descriptive statistics and narrative thematic analysis. In line with the objectives of this scoping review, the included studies were grouped based on the conceptual and methodological characteristics of their interventions related to the MNS and pain contexts. Specifically, the synthesis identified three main domains: (i) clinical interventions based on mirror therapy or mirror-based visual feedback, (ii) interventions based on action observation and motor imagery, and (iii) pain type and context, including phantom limb pain, CPRS, neuropathic pain, and musculoskeletal pain conditions.

## 3. Results

### 3.1. Selection of Sources of Evidence

**Figure 2:**
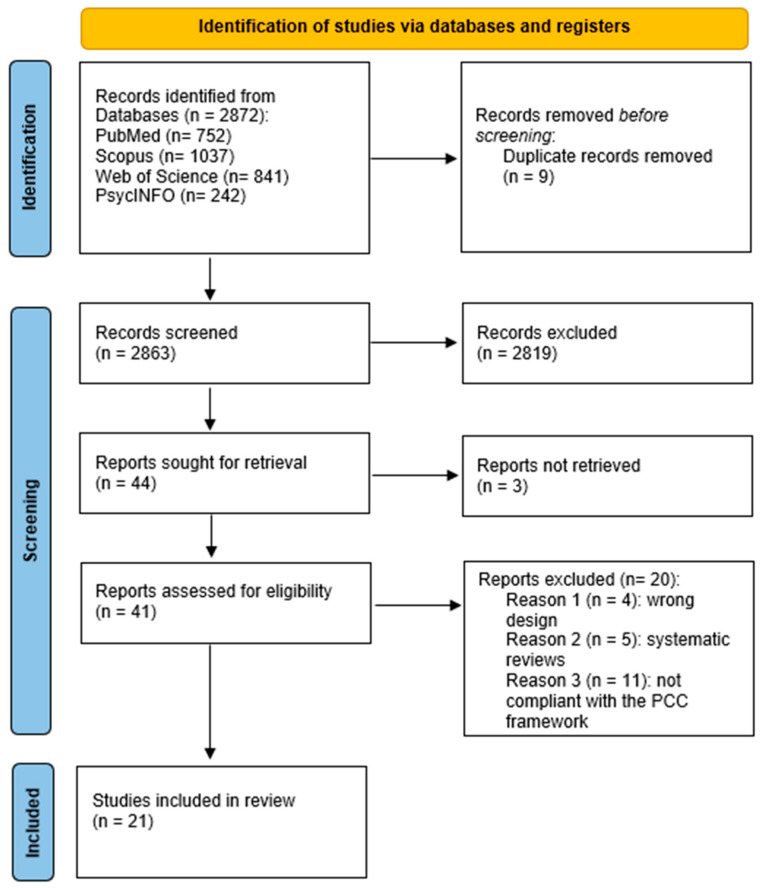
PRISMA flowchart of the selection process.

The search in the databases identified a total of 2872 records (PubMed: 752; Scopus: 1037; Web of Science: 841; PsycINFO: 242). After the removal of 9 duplicates, 2863 records were screened for title and abstract. Of these, 2819 were excluded because they were not relevant to the PCC criteria. 44 full texts were therefore requested for the evaluation of eligibility; however, 3 items were not recovered. In total, 41 articles were evaluated in full text. Of these, 20 were excluded for the following reasons: inappropriate study design (*n* = 4), presence of systematic reviews (*n* = 5), and lack of consistency with PCC criteria (n = 11). Finally, 21 studies were included in the scoping review [20]. The selection process is shown in the PRISMA flowchart (Figure 2).

### 3.2. Characteristics of Sources of Evidence

The populations analyzed consist mainly of adult patients suffering from chronic pain conditions, with a prevalence of studies focused on phantom limb pain in amputees [21,22,23,24,25,26,27,28,29,30,31,32]. Other studies included patients with complex regional pain syndrome [33,34,35], neuropathic pain secondary to nerve injury [36], and chronic or post-procedural musculoskeletal pain [37,38,39].

As for mirror neuron (MNS)-related constructs, these have been operationalized predominantly through mirror therapy or mirror visual feedback interventions [20,21,22,23,24,25,26,27,28,29,30,31,32,33,34,35,36,39,40], while a smaller number of studies have adopted action observation and motor imagery paradigms [37,38]. The interventions were mainly supplied in clinical rehabilitation settings, both outpatient and home-based, with a considerable variability in duration, frequency, and methods of administration. The outcomes assessed primarily included clinical measures of pain intensity, such as the Visual Analogue Scale (VAS) and Numerical Rating Scale (NRS), accompanied or not by functional, behavioral, or quality of life measures. Overall, mirror therapy emerged as the predominant MNS-related intervention, particularly in chronic pain conditions, whereas experimental paradigms directly targeting mirror neuron activity were largely absent (Table 1).

To further clarify the conceptual and methodological heterogeneity of the included studies, a mapping of MNS-related constructs, paradigms, and evidence gaps is presented in Table 2.

### 3.3. Results of Individual Sources of Evidence

The included evidence sources examined interventions attributable to the mirror neuron system in relation to pain, using different clinical and experimental paradigms [20,21,22,23,24,25,26,27,28,29,30,31,32,33,34,35,36,37,38,39,40]. Most studies evaluated mirror therapy or mirror visual feedback interventions applied to populations with chronic pain, particularly individuals with phantom limb pain and CPRS [21,22,23,24,25,26,27,28,29,30,31,32,33,34,35]. In these studies, pain intensity was mainly assessed using self-reported clinical scales, such as the VAS or the NRS, comparing the intervention with control conditions or conventional rehabilitation approaches [21,22,23,24,25,26,27,28,29,30,31,32,33,34,35].

Some studies evaluated mirror therapy in combination with other rehabilitation or neuromodulatory interventions, including exercise programs, relaxation techniques, or non-invasive brain stimulation, and reported outcomes related to pain and, in some cases, functional measures [21,36].

Results were generally presented as changes in clinical outcomes over time or as between-group differences, without allowing direct comparisons between the different components of the interventions. A smaller number of studies adopted action observation and motor imagery paradigms, mainly in patients with chronic musculoskeletal pain, such as chronic neck pain [37,38]. In these studies, outcomes included pain intensity and pressure pain threshold, assessed immediately after the intervention or in the short term [27,38].

## 4. Discussion

Unlike previous reviews focusing on specific interventions or diseases [41], this scoping review maps how the MNS construct itself has been operationalized in pain research across experimental, social, and clinical contexts. In relation to the review question, interventions have been applied across different clinical pain conditions and methodological approaches, with areas that are well explored and others that remain insufficiently investigated. The main finding is that the existing evidence is predominantly focused on clinical and rehabilitative interventions, particularly mirror therapy and mirror visual feedback, rather than on direct experimental investigations of mirror neuron activity. Importantly, in the majority of the studies included, MNS involvement is not directly measured, but inferred indirectly from behavioral outcomes or clinical responses to mirror-based interventions. Neurophysiological or neuroimaging markers typically associated with MNS activities, such as EEG mu rhythm suppression or task-based fMRI activation, are rarely employed. As a result, the link between observed analgesic effects and mirror neuron mechanisms remains largely theoretical or inferential, limiting causal interpretation.

Although the MNS has been extensively discussed in social and affective neuroscience, particularly in relation to pain empathy and embodied simulation [10,17], these conceptual frameworks are rarely translated into empirical pain studies using direct neurophysiological or neuroimaging markers. Instead, MNS-related mechanisms are typically inferred indirectly through clinical interventions based on visual–motor feedback or action observation, consistent with early theoretical accounts of perception–action coupling [4,5,6]. Notably, this imbalance does not reflect a secondary theoretical role of experimental and social neuroscience evidence, highlighting a disconnect between foundational neuroscience models and their empirical application in pain research. Therefore, while experimental and social neuroscience studies have been central in shaping theoretical interpretations of MNS-related mechanisms, their contribution to pain research remains largely indirect, with limited integration into study designs, outcome measures, or mechanistic testing. This gap underscores the need for future research explicitly bridging experimental paradigms and clinical pain investigations.

Within the clinical domain, mirror therapy emerged as the most frequently studied paradigm. Across different settings and populations, several randomized and quasi-experimental studies reported reductions in pain intensity, particularly in phantom limb pain and type I CRPS [21,22,23,24,25,26,27,28,29,30,31,32,33,34,35]. However, findings were not uniform, and some well-designed trials failed to demonstrate clinically meaningful differences compared with control conditions or alternative interventions [25,35]. The failure of mirror therapy or mirror visual feedback to produce analgesic effects may arise from multiple, partially overlapping factors. They include, for instance, heterogeneity in intervention protocols, variability in patient characteristics, differences in pain etiology and chronicity, and limited engagement with the approach. Contextual factors and expectancy effects—mediated by cognitive and affective brain mechanisms—are well-established modulators of pain perception and clinical outcomes, independent of specific treatment mechanisms such as mirror neuron activation (e.g., placebo, clinician behavior, and verbal suggestion). A body of evidence suggests that several mechanisms, such as attentional modulation, multisensory integration, and expectation-driven brain responses, contribute substantially to analgesic outcomes [42,43,44]. Experimental evidence indicates that responses to observed pain are shaped by self-related and contextual factors, with key regions such as the anterior mid-cingulate cortex supporting expectancy, salience, and cognitive evaluation in addition to nociceptive processing [45]. Furthermore, studies using images of pain indicate that empathic responses can be elicited even in the absence of clear action-related or sensorimotor cues [46]. This evidence suggests that modulatory effects on pain arise from higher-order contextual and relational mechanisms (i.e., integrative, network-level frameworks of pain modulation) rather than from direct mirror neuron activation.

Action observation and motor imagery paradigms were explored in a smaller number of studies, mainly in chronic musculoskeletal pain conditions [37,38]. These studies suggested short-term hypoalgesia and increased pain thresholds, aligning with experimental evidence on sensorimotor engagement and expectation-driven modulation of pain [8,9]. However, the limited number of studies and the absence of long-term follow-up prevent firm conclusions regarding their sustained clinical relevance.

Overall, the evidence mapped in this review reveals a clear conceptual gap between the theoretical richness of MNS-related models and their empirical application in pain research. Although the language of mirror neurons, embodied simulation, and sensorimotor resonance is frequently employed, operational definitions and methodological approaches remain heterogeneous and often implicit. As a result, the MNS is commonly invoked as a broad explanatory framework rather than being operationalized as a testable and falsifiable neurobiological mechanism. For example, neuroimaging studies showed that observing pain-related facial expressions activates a distributed network overlapping with regions involved in first-hand pain processing and affective appraisal. However, this overlap reflects shared network-level engagement at the spatial resolution of fMRI, rather than direct evidence of identical neural populations or mirror-specific mechanisms underlying pain empathy or recognition [45].

Across studies, MNS-related terminology is used to describe diverse and partially overlapping phenomena—ranging from empathy and attention to expectancy and multisensory integration—without clear specification of the underlying neural processes or measurable markers. This lack of operational precision constrains mechanistic inference, reduces comparability across studies, and ultimately affects the translation of findings into coherent and clinically meaningful frameworks.

From a broader perspective, the role of the MNS in pain research should be interpreted in light of its theoretical evolution over the past two decades. Early models emphasized mirror neurons as discrete neural substrates supporting action understanding and embodied simulation [5,6]. However, more recent frameworks have progressively shifted attention toward distributed, network-level processes underlying perception–action coupling, expectation, and contextual modulation of pain [47,48]. In this context, the relative decline of studies explicitly testing mirror neuron activity in pain paradigms does not necessarily indicate waning interest, but rather reflects a conceptual reframing of MNS-related ideas within predictive processing and integrative brain models. These approaches emphasize how prior beliefs, contextual information, and multisensory integration shape pain experience, often without requiring strict attribution to mirror neuron activation. This view aligns with critical accounts that challenge classical mirror neuron interpretations and emphasize alternative, distributed explanatory mechanisms [17]. Consequently, contemporary pain research increasingly situates MNS-related concepts as part of broader explanatory frameworks rather than as isolated or dominant mechanisms.

### 4.1. Limitations

This scoping review has several limitations. First, only studies published in English and indexed in selected databases were included, potentially excluding relevant evidence from other sources. Second, consistent with PRISMA-ScR recommendations, no formal assessment of methodological quality or risk of bias was performed; therefore, the strength of evidence supporting individual interventions cannot be inferred. Third, the included studies were highly heterogeneous in terms of design, populations, interventions, and outcomes, which limited the ability to perform quantitative synthesis or direct comparisons.

In addition, although the review aimed to capture experimental, social, and clinical perspectives, the included evidence was largely confined to clinical rehabilitation studies. As a result, experimental neuroimaging and neurophysiological investigations of mirror neuron activity in pain contexts were underrepresented, reflecting a gap in the literature rather than a limitation of the review process itself.

### 4.2. Implications for Practice

From a clinical and nursing perspective, the findings of this scoping review suggest that MNS-related interventions, particularly mirror therapy and action observation-based approaches, represent low-cost, non-invasive, and potentially scalable strategies for pain management, especially in chronic pain conditions such as phantom limb pain. These interventions can be integrated into multidisciplinary pain management programs and, in some cases, delivered in home-based or self-managed formats [26,27].

However, the current evidence base does not support a one-size-fits-all application. Variability in patient characteristics, pain etiology, and intervention protocols underscores the need for individualized care planning and close monitoring of outcomes. For nursing practice, this highlights the importance of patient education, adherence support, and contextual factors such as therapeutic alliance and expectation management, which are known to influence pain outcomes [2,3,9].

Moreover, the conceptual overlap between MNS-related frameworks and broader constructs such as empathy, attention, and expectancy should not be interpreted as evidence that nursing practice operates through mirror neuron mechanisms. These constructs highlight well-established contextual and relational factors known to influence pain perception and clinical outcomes [49]. From a nursing perspective, contributions to pain management primarily arise from patient education, therapeutic alliance, expectation management, and support of adherence to treatment, rather than from any specific neurobiological mechanism related to mirror neuron activity. Framing these effects in contextual and relational terms avoids mechanistic oversimplification and remains consistent with current evidence on pain modulation.

## 5. Conclusions

This scoping review provides a comprehensive overview of how mirror neuron-related frameworks have been applied in pain research. The mapped evidence indicates that, despite extensive theoretical discussion of the MNS in neuroscience, empirical applications in pain research are predominantly clinical and centered on mirror therapy-based interventions. While several studies report beneficial effects on pain outcomes, substantial heterogeneity in conceptualization, methodology, and reporting limits the comparability and generalizability of findings.

Future research should aim to bridge the gap between theoretical models and clinical practice by employing clearer operational definitions, incorporating direct neurophysiological or neuroimaging markers when feasible, and designing longitudinal studies with standardized outcomes. Such efforts may help clarify the role of mirror neuron-related mechanisms in pain modulation and support their integration into evidence-based, patient-centered pain management strategies.

## Figures and Tables

**Table 1:** Characteristics of the included studies.

Author, Year, Country	Study Aims and Design	Population (n; Health Status)	MNS Construct (Definition/Operationalization)	Paradigm (Experimental/Clinical)	Type and Context of Pain	Outcomes Assessed	Main Findings	Reported Limitations
Abolfazli et al., 2019, Iran [20]	To evaluate the early effect of mirror visual feedback after hand surgery; RCT	n = 40; Adult Post-Hand Reconstructive Surgery	Mirror visual feedback as a visual-motor activation of the mirror system	Mirror therapy in clinical rehabilitation	Post-operative pain	Clinical (McGill Pain Questionnaire), functional	Improvement in pain and function in the MVF group compared to the control group	Small sample; Short follow-up
Al Shrbaji et al., 2023, Jordan [37]	Effect of a single action observation session in neck pain; RCT	n = 60; adults with chronic neck pain	Action observation is explicitly based on the mirror neuron system	Observation of movements vs. landscape	Chronic musculoskeletal pain	Clinical (VAS), Behavioral (PPT)	Pain reduction and increased pain threshold after AO	Single session intervention; No follow-up
Brunelli et al., 2023, Italy [21]	Compare MT + PMR vs. MT + relax vs. PT in PLP; RCT	n = 30; amputees with PLP	Mirror therapy as visual feedback for cortical reorganization	Clinical mirror therapy	Chronic neuropathic pain (PLP)	Clinical (BPI, PEQ)	Major pain benefits with PMR + MT	Limited sample; Short follow-up
Cacchio et al., 2009, Italy [33]	Evaluate MT in post-stroke CRPS-I; Placebo RCT	n = 48; post-stroke with CRPS-I upper limb	Mirror visual feedback to modulate sensory-motor maps	MT vs. Mirror Covered	Chronic Pain CRPS-I	Clinical (VAS), functional	Pain reduction only in the MT group	Specific population; Limited generalizability
McCabe et al., 2002, UK [34]	Explore MVF in CRPS-I; controlled pilot study	n = 8; CRPS-I upper limb	Mirror visual feedback to modulate body perception	Experimental MVF	Early/intermediate CRPS pain	Clinical (VAS)	Analgesic effect in the early stages	Very small sample; pilot design
Noureen et al., 2024, Pakistan [22]	Compare MT vs. PT in PLP; Single-blind RCT	n = 60; amputees with PLP	MT as mirror visuomotor activation	Rehabilitation TM	Chronic PLP	Clinical (NRS, TAPES)	Pain Improvement and Prosthetic Fit with TM	Single core; Limited follow-up
Ol et al., 2018, Cambodia [23]	To evaluate MT ± tactile stimulation in mine amputees; RCT	n = 60; PLP and stump pain	Mirror feedback for sensory integration	Clinical TM	PLP and stump pain	Clinical (VAS)	Pain reduction in the TM group	Specific context; Limited resources
Özdemir et al., 2024, Turkey [35]	MT added to rehab in CRPS-I hand; RCT	n = 34; Post-traumatic CRPS-I	Visual Mirror Feedback	MT + rehab	Chronic CRPS-I	Clinical (NRS)	No significant differences between groups	Small sample; short duration
Purushothaman et al., 2023, India [24]	Pre-emptive MT to prevent PLP; RCT	n = 50; BK amputation	Early mirror feedback to prevent maladaptive reorganization	Preventive MT	Post-operative PLP prevention	Clinical (incidence and NRS PLP)	Lower incidence and intensity of PLP in the MT group	Limited follow-up; Single center
Rothgangel et al., 2018, Netherlands [25]	Traditional MT vs. AR-MT vs. usual care (PACT); multicentre RCT	n = 75; Chronic PLP	Traditional and AR visual mirror feedback	MT/AR-MT clinical	Chronic PLP	Clinical (intensity, duration), functional	No clinically relevant differences between groups	Variable adherence; possible placebo effect
Yun & Kim, 2019, Korea [40]	Evaluate MT in mutilating hand injuries; RCT	n = 30; mutilating hand injuries	Mirror therapy for sensory-motor reorganization	MT + PT	Traumatic upper limb pain	Clinical (VAS), functional	Improvement of pain and function with TM	Small sample; short follow-up
Yildirim & Kanan, 2016, Turkey [26]	MT in the management of PLP; quasi-experimental	n = 15; amputees with PLP	Mirror feedback as visual limb replacement	Clinical TM	Chronic PLP	Clinical (Numeric Pain Intensity Scale)	Progressive reduction in PLP over 4 weeks	No control; Small sample
Ferreira et al., 2020, Brazil [36]	To evaluate the effect of tDCS associated with mirror therapy in post-avulsion neuropathic pain of the brachial plexus; Pilot Double-Blind RCT	n = 16; patients with brachial plexus avulsion and neuropathic pain	Mirror therapy as visual-motor feedback to modulate cortical reorganization	tDCS + MT vs. sham + MT	Chronic neuropathic pain	Clinical (VAS), functional	Greater pain reduction in the tDCS + MT group compared to the control	Small sample; pilot study; short follow-up
Suso-Martí et al., 2019, Spain [38]	To evaluate the effect of motor imagery and action observation on hypoalgesia in chronic neck pain; Single-blind RCT	n = 42; patients with chronic neck pain	Action observation and motor imagery as activation of the mirror neuron system	AO/MI of therapeutic exercises vs. placebo	Chronic musculoskeletal pain	Clinical (VAS), Behavioral (PPT)	AO/MI induce hypoalgesia and increased pain threshold compared to control	Short-term intervention; No follow-up
Shariaty & Taheri, 2024, Iran [27]	To evaluate the efficacy of home mirror therapy in PLP; RCT	n = 40; below-the-knee amputees with PLP	Mirror therapy as visual limb replacement for sensory-motor reorganization	Home-based MT vs. usual care	Chronic Phantom limb pain	Clinical (VAS)	Significant reduction in PLP intensity in the MT group	Home self-management; Possible variability of adherence
Martín Pérez et al., 2024, Spain [39]	To evaluate MT on post-needling pain in lateral elbow pain; Pilot Prospective Controlled Study	n = 44; patients with lateral epicondylalgia	Mirror visual feedback to modulate the perception of movement and pain	MVF after dry needling	Acute iatrogenic pain	Clinical (VAS), functional	Lower post-procedure pain intensity in the MT group	Pilot study; acute, non-chronic pain; short follow-up
Finn et al., 2017, USA [28]	To evaluate the efficacy of mirror therapy in upper limb PLP; RCT	n = 15; male amputees with upper limb PLP	Mirror therapy as a visual-motor activation of the mirror system	MT vs. Mirror Covered/Imagery	Chronic Phantom limb pain	Clinical (VAS), PLP frequency/duration	Improvement in PLP intensity and duration in the MT group	Small sample; Males only
Darnall & Li, 2012, USA [29]	To evaluate the feasibility and preliminary effects of home TM in PLP; Pilot prospective study	n = 40; amputees with PLP	Mirror therapy as self-administered visual feedback	Home-based self-delivered MT	Chronic Phantom limb pain	Clinical (NRS), sleep quality	Pain reduction and improved sleep after TM	Absence of control group; pilot design
Anghelescu et al., 2016, USA [30]	Describe the incidence/duration of PLP and compare pediatric patients treated with MT + standard care vs. standard care; Comparative retrospective study	n = 21; Children/adolescents and young adults with oncological amputation	Mirror therapy as visual-motor feedback to reverse the maladaptive reorganization of the sensory-motor system	Mirror therapy in the pediatric clinical setting	Phantom limb pain in pediatric oncology	Clinical: PLP incidence, PLP duration, pain intensity, analgesic use	MT is associated with a lower incidence of PLP at 1 year and shorter duration of PLP compared to the non-MT group	Retrospective drawing; small sample; non-randomized; possible therapeutic confounding
Anaforoğlu Külünkoğlu et al., 2019, Turkey [31]	Compare MT vs. phantom exercises on pain, QoL, and psychological status in PLP; Prospective RCT	n = 40; unilateral transtibial amputees with chronic PLP	MT as a visual illusion to modulate cortical reorganization; PE based on mental imagery	MT vs. Phantom Exercises for 4 weeks + 3- and 6-month follow-up	Chronic Phantom limb pain	Clinical (VAS), QoL (SF-36), Psychological Status (BDI)	Both improve, but MT is more effective than PE on pain intensity and BDI; better QoL outcomes in the MT group	Limited sample; impossibility of blindness; Variable home adherence
Mallik et al., 2020, India [32]	Compare relative benefits of MT vs. mental imagery in PLP; Prospective non-blinded RCT	n = 92; amputees with PLP in rehabilitation	MT as visual feedback for cortical reorganization; mental imagery as imaginative activation of motor circuits	MT vs. Mental imagery + conventional rehab	Chronic Phantom limb pain	Clinical: VAS at baseline, 4, 8, 12 months	Significant reduction in pain in both groups, but more effective MT than mental imagery (VAS 7 to 2.7 vs. 5.9)	Not blind; only clinical assessment of pain; absence of functional outcomes

Abbreviations: AO, Action Observation; AR-MT, Augmented Reality Mirror Therapy; BDI, Beck Depression Inventory; BPI, Brief Pain Inventory; CRPS, Complex Regional Pain Syndrome; CRPS-I, Complex Regional Pain Syndrome Type I; EEG, Electroencephalography; fMRI, Functional Magnetic Resonance Imaging; MI, Motor Imagery; MNS, Mirror Neuron System; MT, Mirror Therapy; MVF, Mirror Visual Feedback; NRS, Numeric Rating Scale; PACT, Prosthesis-Adapted Computer-based Training; PEQ, Prosthesis Evaluation Questionnaire; PLP, Phantom Limb Pain; PMR, Progressive Muscle Relaxation; PPT, Pressure Pain Threshold; PT, Physical Therapy; QoL, Quality of Life; RCT, Randomized Controlled Trial; SF-36, Short Form Health Survey (36 items); TAPES, Trinity Amputation and Prosthesis Experience Scales; tDCS, Transcranial Direct Current Stimulation; VAS, Visual Analogue Scale.

**Table 2:** Conceptual and methodological mapping of mirror neuron system-related approaches in pain research.

Conceptual Domain	MNS-Related Construct	Paradigm or Intervention	Level of Inference on MNS	Pain Type or Context	Outcomes Assessed	Main Evidence Gaps
Clinical rehabilitation	MT/MVF	Visual–motor illusion using mirror feedback	Indirect	Chronic pain, mainly phantom limb pain and CRPS	Pain intensity (e.g., VAS, NRS), functional measures, QoL	Absence of direct neurophysiological or neuroimaging markers of MNS activity; heterogeneity of protocols
Clinical rehabilitation	Action observation	Observation of goal-directed movements	Indirect	Chronic musculoskeletal pain	Pain intensity, PPT	Short-term outcomes; limited follow-up; small number of studies
Clinical rehabilitation	MI	Mental simulation of movement	Indirect	Chronic musculoskeletal pain	Pain intensity, PPT	Difficulty isolating MI-specific effects; variability in training protocols
Experimental or social neuroscience	Pain empathy	Observation of others in pain	Theoretical or inferential	Experimental pain, social contexts	Neural activation patterns, behavioral responses	Limited translation into clinical pain interventions
Experimental neuroscience	Sensorimotor resonance	Neurophysiological or neuroimaging proxies (e.g., EEG mu suppression, fMRI activation)	Indirect or proxy-based	Experimental paradigms	Neural markers of sensorimotor engagement	Rarely used as primary outcomes in clinical pain studies
Translational approaches	Combined or augmented interventions (e.g., MT + neuromodulation)	MT combined with adjunctive techniques	Indirect	Neuropathic and chronic pain	Pain intensity, functional outcomes	Small samples; pilot designs; lack of mechanistic clarification

Abbreviations: MT, mirror therapy; MVF, mirror visual feedback; CRPS, Complex Regional Pain Syndrome; MNS, mirror neuron system; MI, motor imagery; QoL. Quality of life; PPT, pressure pain threshold; EEG, Electroencephalography; fMRI, Functional Magnetic Resonance Imaging.

## Data Availability

No new data were created or analyzed in this study. Data sharing does not apply to this article.

## Supplementary Materials

The following supporting information can be downloaded at: https://www.mdpi.com/article/10.3390/healthcare14020280/s1. Supplement S1 contains the search string of the consulted databases.
