# Supplementary material for: Mirror Neurons and Pain: A Scoping Review of Experimental, Social, and Clinical Evidence
Source: Healthcare (Basel). 2026 Jan 22;14(2):280. doi: 10.3390/healthcare14020280 (PMC12841073; doi:10.3390/healthcare14020280)
Supplement: Supplementary file 1 [file healthcare-14-00280-s001.zip › healthcare-4103333-supplementary.pdf]

Supplement S1: search string of the consulted database

Database	String	Results
PubMed	("Mirror Neurons"[MeSH] OR "mirror neuron*"OR "mirror neuron system" OR "action observation" OR "motor imagery" OR "mirror therapy"OR "mirror visual feedback"OR "embodied simulation") AND (“Pain"[MeSH] OR "Nociception"[MeSH] OR pain OR nocicept* OR analges* OR "pain empathy" OR "empathy for pain")	752
SCOPUS	(TITLE-ABS-KEY ("mirror neuron*" OR "mirror neuron system" OR "action observation" OR "motor imagery" OR "mirror therapy" OR "mirror visual feedback" OR "embodied simulation") AND TITLE-ABS-KEY (pain	1037

	OR nocicept* OR analges* OR "pain empathy" OR "empathy for pain"))	
Web of Science	TS=("mirror neuron*" OR "mirror neuron system" OR "action observation" OR "motor imagery" OR "mirror therapy" OR "mirror visual feedback" OR "embodied simulation") AND TS=(pain OR nocicept* OR analges* OR "pain empathy" OR "empathy for pain")	841
PsycINFO	("mirror neuron*" OR "mirror neuron system" OR "action observation" OR "motor imagery" OR “mirror therapy" OR "mirror visual feedback" OR "embodied simulation") AND (pain OR nocicept* OR analges* OR "pain empathy" OR "empathy for pain")	242
